# Outer Membrane Vesicles (OMVs) of *Pseudomonas aeruginosa* Provide Passive Resistance but Not Sensitization to LPS-Specific Phages

**DOI:** 10.3390/v14010121

**Published:** 2022-01-11

**Authors:** Daria Augustyniak, Tomasz Olszak, Zuzanna Drulis-Kawa

**Affiliations:** Department of Pathogen Biology and Immunology, University of Wroclaw, Przybyszewskiego 63/77, 51-148 Wroclaw, Poland; daria.augustyniak@uwr.edu.pl (D.A.); tomasz.olszak@uwr.edu.pl (T.O.)

**Keywords:** *Pseudomonas aeruginosa*, outer membrane vesicles (OMV), lytic phages, resistance to phages, sensitization to phages, passive protection

## Abstract

Outer membrane vesicles (OMVs) released from gram-negative bacteria are key elements in bacterial physiology, pathogenesis, and defence. In this study, we investigated the role of *Pseudomonas aeruginosa* OMVs in the anti-phage defence as well as in the potential sensitization to LPS-specific phages. Using transmission electron microscopy, virion infectivity, and neutralization assays, we have shown that both phages efficiently absorb on free vesicles and are unable to infect *P. aeruginosa* host. Nevertheless, the accompanying decrease in PFU titre (neutralization) was only observed for myovirus KT28 but not podovirus LUZ7. Next, we verified whether OMVs derived from wild-type PAO1 strain can sensitize the LPS-deficient mutant (Δ*wbpl* PAO1) resistant to tested phages. The flow cytometry experiments proved a quite effective and comparable association of OMVs to Δ*wbpl* PAO1 and wild-type PAO1; however, the growth kinetic curves and one-step growth assay revealed no sensitization event of the OMV-associated phage-resistant *P. aeruginosa* deletant to LPS-specific phages. Our findings for the first time identify naturally formed OMVs as important players in passive resistance (protection) of *P. aeruginosa* population to phages, but we disproved the hypothesis of transferring phage receptors to make resistant strains susceptible to LPS-dependent phages.

## 1. Introduction

Outer membrane vesicles (OMVs) released by gram-negative bacteria are proteoliposomal nanoparticles that play an important role in bacterial physiology, pathogenesis, intraspecies or interspecies interactions, as well as mammals interactions [1,2]. In host-pathogen interactions during infection, OMVs may serve as enhancers of microbial adherence and biofilm formation [3,4,5], inductors of inflammation and apoptosis [6,7,8,9], and as inhibitors of host immune response [10,11,12].

In microbial communities, OMVs may be used by bacteria as an offensive or defensive weapon. Concerning the former, OMVs can deliver bactericidal toxins or enzymes to other bacteria [13,14] as well as enzymes, DNA, and small RNA to mammalian cells, causing their injury [15]. In the defensive aspect, *Moraxella catarrhalis* OMVs carrying β-lactamases confer protection to the producer and accompanying *Streptococcus pneumoniae* against β-lactam antibiotics [16]. It has been also shown that OMVs are involved in the trapping of antibiotics, cationic peptides, or serum complement, thus providing cross-resistance and increasing virulence among bacteria or pathogenic yeasts [17,18]. Recent studies show that OMVs can participate in bacteria–phage interplay, by being a phage decoy and sequestering phage particles as first documented for OMVs of *E. coli* and phage T4 [19]. Furthermore, membrane vesicles (MV) derived from gram-positive *Bacillus* genera can mediate the exchange of cell surface components, including phage receptors, thus facilitating sensitization to phages [20].

*Pseudomonas aeruginosa* is one of the most life-threatening pathogens due to its high intrinsic drug resistance and genome plasticity that condition high adaptation to adverse environmental changes and promote survival and persistence at different stages of pathogenesis [21,22]. These bacteria cause opportunistic infections, including burn wound infections, urinary tract infections, keratitis, otitis externa, and respiratory tract infection. *P. aeruginosa* strains possess a huge arsenal of virulence properties/factors, including production of alginate, biofilm formation, pyoverdine siderophore, lipopolysaccharide (LPS), quorum sensing (QS), type 4 pili (T4P), the type II and III and VI secretion systems, lipases, proteases (AprA and PIV), elastases (LasA and LasB), urease, and exotoxin A [22,23,24]. Many of these virulence factors have been identified as OMV elements [25]. OMVs can be released by planktonic or sessile cells by PQS (*Pseudomonas quinolone signal*)-induced mechanism or cell lysis, significantly increasing the virulence capacity or contributing to virulence-associated processes [26]. *P. aeruginosa* OMVs can (i) prime host tissue surfaces for bacterial adhesion [5], (ii) facilitate the removal of competing bacteria from the environment during infection, as well as (iii) reduce CFTR (cystic fibrosis transmembrane conductance regulator) Cl^−^ secretion from cystic fibrosis bronchial epithelial cells, thus reducing the bacterial clearance from the lungs [13,27].

The use of phages for the treatment of bacterial infections has been extensively studied as an alternative therapeutic strategy [28,29,30]. Since *P. aeruginosa* is one of the leading opportunistic pathogens involved in hospital-acquired infections, large fractions of the phage application studies and genome-driven phage–bacteria interplay projects are focused on this bacterium [31,32,33,34]. To our knowledge, there are no reports published yet deliberating the role of *P. aeruginosa* OMVs in fighting phages. In general, two groups of defence strategies against phage infection can be recognized. 

The first one is triggered in the presence of a phage genome inside the cell (cellular interior). These mechanisms are exemplified by bacterial expression of anti-phage defences cutting phage DNA, including innate systems (restriction-modification, BREX, DND, DISARM, etc.) as well as adaptive ones, such as CRISPR–Cas (clustered regularly interspaced short palindromic repeats-CRISPR associated proteins), destroying the invader and remembering it [35]. The phage life cycle can be inhibited also by the chemical defence, viperins, retrons, and cell signalling coupled with abortive infection leading to cell death before completion of phage reproduction and many other mechanisms that have not yet been fully elucidated [35,36,37,38]. 

The second strategy is the primary mechanism to avoid phage infection, and it is based on blocking phage adsorption to the bacterial cell. That purpose may be achieved by the modification or alteration of surface receptors by (i) mutation or deletion of genomic DNA encoding receptor that leads to receptor alteration/loss, (ii) masking its binding site, (iii) and regulation of receptor expression by lysogenic phages as well as (iv) global regulatory pathways [34,39,40]. Since OMVs released from bacterial cells are equipped with numerous surface macromolecules, such as LPS, type IV fimbriae, outer membrane proteins (OMPs), and capsular polysaccharides (CPS) [37,41], their contribution to potential phage receptor transfer and alteration is also very likely. Such phenomenon of phage protection by OMVs has already been shown in *Salmonella* and *Vibrio cholerae* models [41,42].

That enormous (extraordinary) adaptive capability that enables *P. aeruginosa* to survive various hostile conditions, such as inhabiting various environmental niches, host invasion, or phage exposure, constitute an axis of interconnection, the understanding of which is of great importance. The goal of the study was to extend the OMV-mediated phage-protection paradigm to an important human opportunistic pathogen and characterize the role of *P. aeruginosa* OMVs in bacteria–phage interplay and anti-phage defence in the PAO1 strain model. We verified two main hypotheses of OMVs phage-bacteria interactions first as a protective barrier and secondly as a sensitization element to LPS-dependent phage infection.

## 2. Materials and Methods

### 2.1. Reagents

TSB (Tryptone Soya Broth, OXOID, Hampshire, UK); TSA (Tryptone Soya Agar, OXOID, Hampshire, UK); Bradford reagent (Protein Assay Dye Reagent Concentrate, Bio-Rad, München, Germany); Gelcode Blue stain reagent (Thermo Scientific, Rockford, IL, USA), and fluorescein isothiocyanate (FITC, ThermoScientific, Rockford, IL, USA) were used.

### 2.2. Bacterial Strain and Phages

The *P. aeruginosa* PAO1 (ATCC 15692) reference strain and its Δ*wbpl* knock-out mutant deficient in the A-band and B-band O-antigens biosynthesis (provided by Andrew M. Kropinski from the Laboratory of Foodborne Zoonoses, Guelph, ON, Canada) were used in this study. Bacteria were stored at −70 °C in TSB supplemented with 20% glycerol. *Pseudomonas* phages KT28 and LUZ7 were propagated as previously described [43]. The phage titre was assessed using the double-agar layer technique. Purified phage samples were stored at 4 °C. Phage characteristics are presented in Table 1.

### 2.3. Outer Membrane Vesicles Isolation

Outer membrane vesicles (OMVs) isolation was adapted according to our previous protocol with few modifications [12]. Briefly, overnight culture of *P. aeruginosa* was diluted 50-fold in 500 mL of TSB and incubated at 37 °C for 16–18 h, with agitation (150 rpm). The culture was harvested by centrifugation (8000× *g* for 15 min at 4 °C). The supernatant was collected and passed through a 0.22-μm-pore size filter vacuum pump (Merck, Millipore, Billerica, MA, USA). The filtrates were concentrated using 100 kDa Vivaspin centrifugal concentrators (Amicon Ultra, Merck Millipore, Cork, Ireland) at 5000× *g* for 30 min at 4 °C. The concentrated supernatants were subsequently pelleted overnight (100,000× *g*, at 4 °C) in an ultracentrifuge (Beckman Coulter Optima L-90K, USA). The pellets containing OMVs were re-suspended in 500 μL of sterile PBS buffer (pH 7.4), aliquoted, and stored at −20 °C. For some experiments, OMVs were subjected to additional centrifugation at 16,000× *g* for 30 min at 4 °C to clear most contaminating flagella [5]. The sterility of the OMV preparations was confirmed on TSA. The protein concentrations in OMV preparations were measured using Bradford assay, and the quality of the OMV samples was confirmed in 12% SDS-PAGE.

### 2.4. OMV Association Assay by Flow Cytometry

OMV association assay was performed as described previously [18] with some modifications.

(i) OMVs’ labelling: Initially, 500 μL OMVs (100 μg/mL) in PBS were concentrated using 30 kDa Vivaspin centrifugal concentrators (Amicon Ultra, Merck Millipore, Cork, Ireland) at 14,000× *g* for 10 min at 4 °C to remove PBS. The collected OMVs were reconstituted with 500 μL of 0.05 M carbonate/bicarbonate buffer (pH 9.5) and washed by centrifugation on Vivaspin as described before. The collected OMVs (~50 μL) were labelled with 500 μL of 1 mg/mL FITC at carbonate/bicarbonate buffer for 30 min at 37 °C with gentle mixing in the dark. The remaining fluorochrome was rinsed 3 times with a 500 μL of cold carbonate/bicarbonate buffer each time, using 30 kDa Vivaspin. The final FITC-labelled OMVs were resuspended in PBS containing at the concentration of 500 μg/mL.
(ii) OMV association with bacteria: 0.5 mL of fresh bacterial culture corresponding to OD_600_ = 0.23–0.25 was centrifuged and subsequently washed with 1 mL of PBS (8000× *g*, 10 min, 4 °C). The pellet was resuspended in 100 μL of OMV-FITC conjugate (from 20 to 320 μg/mL OMVs) supplemented with 5 mM CaCl_2_ and incubated for 3 h at 37 °C with gentle mixing in the dark. Afterwards, samples were washed twice with PBS by centrifugation (8000× *g*, 10 min, 4 °C) to remove free OMVs particles and finally resuspended in 500 μL of PBS.
(iii) Flow cytometric analysis: To detect bacterial cells associated with FITC-labelled OMVs, flow cytometry analysis was performed using GUAVA^®^ EasyCyte flow cytometer (Millipore, Seattle, WA, USA). Before analysis, the samples were diluted at 1:10 to obtain approximately 1–5 × 10^6^ CFU/mL (colony-forming units/mL) in PBS. Fluorescence intensity of bacterial cells associated with OMVs was analysed for green fluorescence in the FL1 channel by collecting 5000 events. Data were expressed as mean fluorescence intensity (MFI). Data analysis was performed using InCyte Merck Guava software (Millipore, Hayward, CA, USA).

### 2.5. Bacterial Growth Assay with Free OMVs and Phages

The overnight TSB culture of *P. aeruginosa* PAO1 was refreshed to early log phase (OD = 0.2, A_600_ nm) by incubation at 37 °C with agitation. The bacteria were centrifuged (8000× *g*, 10 min, 4 °C), washed with saline, adjusted to OD 0.2 and diluted 100×. For testing the lytic activity of phages in the presence of OMVs, the log-phase bacterial suspension (10^5^ CFU/mL) was treated with phages (multiplicity of infection, MOI = 1) and with or without 20 μg/mL of OMVs. Each option was incubated in a water bath at 37 °C in 1% TSB medium (*w*/*v*) in the final volume of 200 μL for 120 min. Every 30 min, the 10 μL aliquots of 10-times diluted bacterial suspensions were plated in triplicate on TSA agar plates. After 18 h of incubation at 37 °C, the colony counts and the CFU/mL were calculated. The lytic activity of phages was expressed in each time point as a decrease in CFU/mL in the reference to uninfected control (time 0). All microbicidal assays were performed at least two times in triplicate.

### 2.6. Phage Neutralization Assays

Then, an 18-h TSB culture of *P. aeruginosa* PAO1 was refreshed to early log phase (OD_600_ = 0.2) by incubation at 37 °C with agitation. The bacteria were centrifuged (8000× *g*, 10 min, 4 °C), washed with saline, adjusted to OD_600_ = 0.2, and diluted 100×. The following test options were prepared: (i) phage inoculum of 10^5^ PFU/mL (plaque-forming units/mL) and 80 μg/mL OMVs and (ii) phage inoculum of 10^5^ PFU/mL. The contents were mixed and incubated in a water bath at 37 °C for 3 h. Every 30 min, the PFU/mL was determined by the double-layer TSA plates method.

### 2.7. Growth Kinetics Measurements after OMV Association

To fuse OMVs with bacteria, 0.5 mL of fresh bacterial culture corresponding to OD_600_ = 0.2 was transferred to a 2-mL Eppendorf tube, centrifuged, and subsequently washed with 1 mL of PBS, pH 7.4 (8000× *g*, 10 min, 4 °C). The pellet was resuspended in 100 μL of OMVs at the final concentration of 320 μg/mL in PBS containing 5 mM CaCl_2_ to facilitate fusion [45] and incubated for 3 h at 37 °C with mixing (200 rpm) in the dark. Afterwards, samples were washed twice with 1 mL of PBS by centrifugation (8000× *g*, 10 min, 4 °C) to remove free OMVs particles and finally resuspended in 0.5 mL of PBS. The bacteria were diluted 50× in TSB to obtain 10^6^ CFU/mL. A hundred µL of bacterial suspension and 100 µL of phages (MOI = 1) were added to each microplate well. All growth kinetics experiments were performed on the flat-bottomed 96-well microplates (Nunclon Delta Surface 167008, Thermo Scientific, Roskilde, Denmark) at 37 °C in the final volume of 200 µL. The growth rate was measured using a Varioskan LUX multimode microplate reader (Thermo Scientific, Vantaa, Finland) with OD_600_ measurements for 24 h at 30 min intervals with agitation (60 rpm). The data for statistical analyses were expressed as AUC (area under the curve).

### 2.8. Lytic Phage Cycle after OMV Association (One-Step Growth Assay)

The bacteria-OMVs-associated population was prepared as described in Section 2.7, and then, 100 µL of bacterial suspension in TSB (10^8^ CFU/mL) was mixed with 100 µL of phages (MOI = 1) and incubated at 37 °C for 90 min. The phage titre (PFU/mL) was monitored every 15 min by the double-layer TSA plates method.

### 2.9. Transmission Electron Microscopy Analyses (TEM)

The OMVs preparation for TEM was adapted from our previous protocol [9]. Briefly, OMVs were visualized by standard negative staining using a formvar-coated copper grid (Christine Gröpl Electronenmikroskopie, Tulln, Austria) and 2% (*w*/*v*) aqueous solution of uranyl acetate. The OMVs were imaged with a TEM operating at an acceleration voltage of 150 kV (Hitachi H-800, Tokyo, Japan) or (Tesla BS 540, Brno, Czech Republic operated at 80 kV. During the purification of phages, the sterile and filtered phage lysate was centrifuged at 25,000× *g*, 60 min, 4 °C. The pellets were washed twice with 500 µL of ammonium acetate (0.1 M, pH 7.0) by centrifugation (parameters as above), and the quality of phage particles was checked using TEM. To visualize the interaction of phages with OMVs, the phage particles in PBS supplemented with 5 mM CaCl_2_ (10^8^ PFU/mL) were mixed with OMVs (100 µg/mL) and incubated for 30 min at 37 °C. Formed OMV-phage complexes were next deposited on carbon-coated 200-mesh Formvar copper grids (Christine Gröpl Electronenmikroskopie, Tulln, Austria), stained with uranyl acetate (2%, pH 4.5), and examined using TEM.

### 2.10. Statistical Analysis

The data were expressed as the mean ± SEM (standard error of the mean). Normality and homogeneity of variance assumptions were checked by Shapiro–Wilk and Levene’s test, respectively. For comparisons between two groups, the *t*-test for independent variables was used. For multiple comparisons, data were analysed using non-parametric Kruskal–Wallis ANOVA rang test. Differences were considered statistically significant if *p* < 0.05 using the Statistica (version 13.1) software (StatSoft, Krakow, Poland).

## 3. Results

### 3.1. Charateristics of P. aeruginosa OMVs

**Figure 1:**
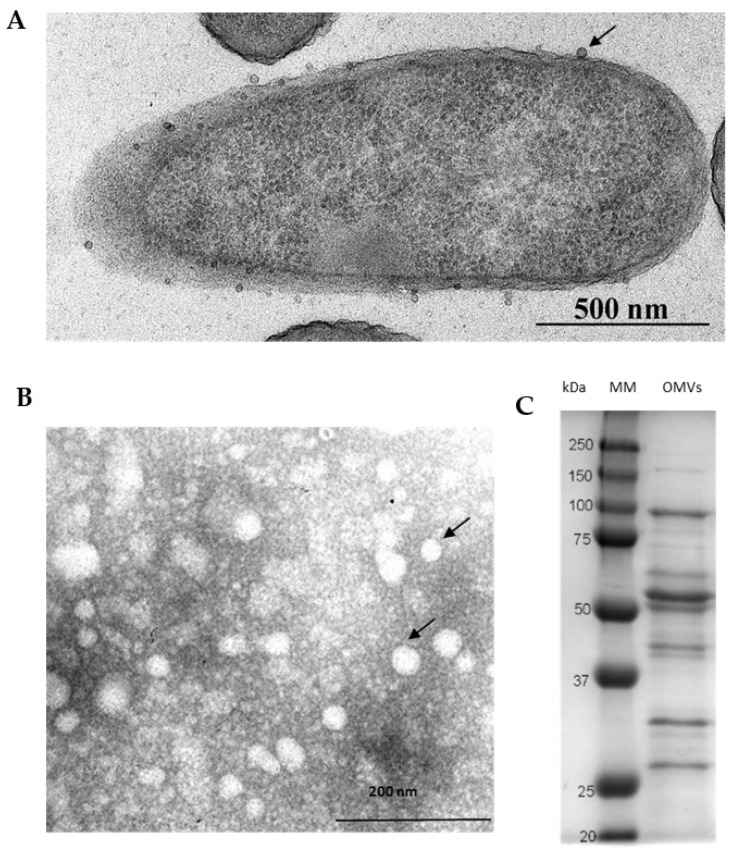
Physical characterization of outer membrane vesicles (OMVs) produced by *P. aeruginosa* PAO1 cells: (**A**) TEM micrograph of *P. aeruginosa* cells releasing OMVs (magnification, ×12,000); (**B**) TEM micrograph of isolated *P. aeruginosa* OMVs, with vesicles indicated by arrows (magnification, ×30,000); (**C**) the representative proteinogram of 12% SDS-PAGE electrophoresis of OMVs; the protein profiles were visualized using Coomassie staining.

The *P. aeruginosa* PAO1 strain was used as an OMV source because of its comprehensive genotypic and phenotypic characterization in the literature and as the host of lytic phages selected for this study. As shown in the transmission electron microscopy (TEM) images, the diameters of OMVs from PAO1 planktonic cells varied between 25–75 nm (Figure 1A,B). The zeta potential of vesicles in miliQ (20 µg/mL) was −26.8 ± 0.71 mV as assessed by the Zeta-sizer Nano-ZS 90 (Malvern, UK). The protein components of OMVs are shown in Figure 1C.

### 3.2. P. aeruginosa Free OMVs Passively Protect against Infection with Phages Recognizing LPS

To evaluate whether OMVs can inhibit phage infection, OMVs obtained from *P. aeruginosa* PAO1 wild-type strain were combined with the PAO1 population treated with LPS-specific phages. Two different lytic phages, a myovirus KT28 (*Pbunavirus*) and podovirus LUZ7, were used as models (Table 1). The selection of phages for the vesicle experiments was not random; both recognise LPS as their receptor, and both are capable of infecting the same host, *P. aeruginosa* PAO1, but not its LPS-deficient Δ*wbpl* mutant. Since phage adsorption to target receptors on the host cell surface is sometimes hindered, most often by envelopes, mucus, and polymeric molecules anchored to the cell wall/membrane, the tail fibres of some phages are equipped with specific virion-associated enzymes (e.g., depolymerases, deacetylases), which pave the way to the final receptor. Analysis of the annotated genomes of phages KT28 and LUZ7 did not unequivocally show the presence of genes encoding depolymerases in either virus. Although the ORF56 of phage LUZ7 contains a hypothetical domain of the GDSL-like lipase/acylhydrolase family, no visible halo zone on the bacterial lawn was detected as a characteristic plaque morphology for depolymerase producing phages. Therefore, we concluded that the taxonomic affiliations and the virion types (LUZ7-podovirus, KT28-myovirus) are the main differences between these phages. The differences in virion structure imply different infection strategies. Podoviruses equipped with a short, noncontractile tail eject their DNA following the rearrangement of structural tail proteins, which create a specific tail extension that serves as a channel for the transfer of genetic material [46]. Myoviruses have a tail composed of a rigid tube surrounded by a contractile sheath. As a result of conformational changes within the baseplate, the sheath contracts, and the rigid tube pierces through the cell envelope, enabling the DNA transfer [47].

**Figure 2:**
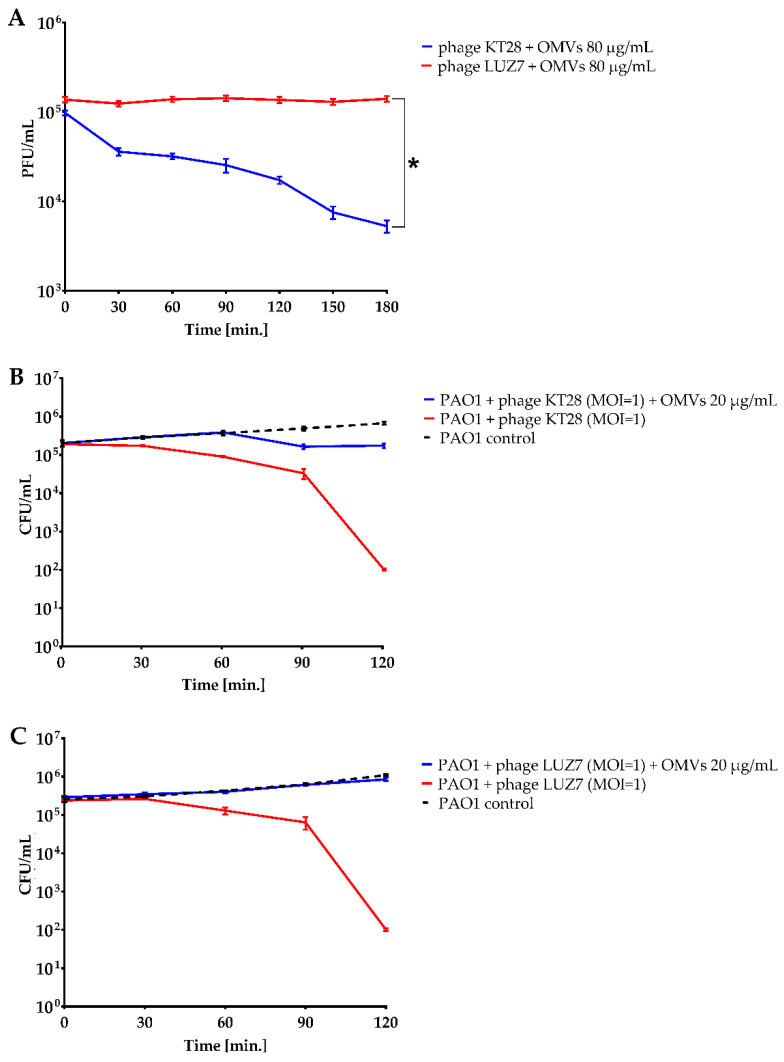
The inhibition of lytic phage activity by OMVs: (**A**) phage particles neutralization assay by OMVs at the concentration of 80 µg/mL; the comparisons of AUC were performed by *t*-test for independent variables; * *p* < 0.0001. (**B**,**C**) The passive protection of *P. aeruginosa* PAO1 cells against lytic phage infection provided by free OMVs at 20 µg/mL. The infection was monitored by the colony count of the surviving population treated with myovirus KT28 (**B**) and podovirus LUZ7 (**C**) at MOI = 1. The phage-uninfected PAO1 culture without the addition of OMVs was considered as the control. The curves were established on the average of at least two independent experiments performed in at least two working replicates, PFU/mL or CFU/mL measurement ± SEM bars.

The phage lytic activity in the presence or absence of OMVs was measured by assessing two independent parameters: (i) PFU count after neutralization of infective virions by free OMVs (Figure 2A) and (ii) bacterial counts of phage-infected culture (Figure 2B,C). In preliminary experiments, the titration of optimal concentrations of OMVs was performed using from 10 μg/mL to 160 μg/mL of vesicles in neutralization assays and from 10 μg/mL to 80 μg/mL of vesicles in passive protection assays. Based on calibration results, the lowest concentration of OMVs that produced the clear effect, i.e., 80 μg/mL (neutralization) and 20 μg/mL (protection), was selected and used in remaining experiments (Supplementary Figure S1).

Evaluating virion neutralization of myovirus KT28 over a one-log reduction (>90%) in PFU/mL was obtained after 3 h of incubation with OMVs. In contrast, phage LUZ7 particles infectivity seemed to be not counteracted by bacterial vesicles, as in the presence of OMVs, the PFU titre remained at the initial level throughout the experiment (Figure 2A). The inconsistency in the neutralization efficacy by LPS deposited on OMV surface might be correlated with the differences in the adsorption mechanisms between myo- and podoviruses.

The passive protection with OMVs was confirmed in the second set of experiments for both cases, showing almost 100% of bacterial survival in the presence of phages and OMVs, whereas phage infected population in the absence of externally applied vesicles was reduced by over 3 logs for KT28 and LUZ7 phages (Figure 2B,C).

**Figure 3:**
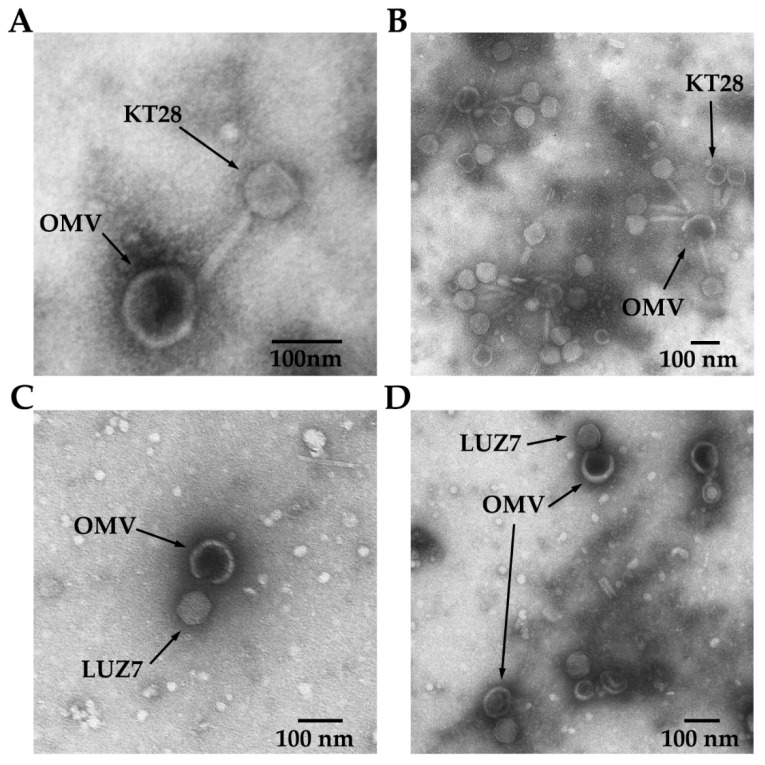
Interactions between OMVs and LPS-dependent phages visualized in TEM technique. (**A**) Adsorption and (**B**) capsid content injection of myovirus KT28; (**C**,**D**) podovirus LUZ7 adsorption lacking capsid content injection into OMVs interior. Magnifications: (**A**) 50,000×, (**B**) 30,000×, (**C**,**D**) 16,000×.

To confirm that the ability of vesicles to interact with LPS-dependent phages is responsible for the mentioned biological effects, we performed an OMV-phage binding assay and visualization by electron microscopy (Figure 3). It turned out that free OMVs effectively bind KT28 particles followed by active injection of capsid content into the vesicle interior (empty capsids and contractile tails), which explains the neutralization event. On the contrary, phage LUZ7 virions were adsorbed on OMVs surface, but it did not result in genetic material transfer into vesicles (lack of neutralization). We may thus conclude that even without a direct neutralization of phage particles, the protective shield made by externally delivered OMVs is provided probably both by sequestrating infective particles and/or by the physical barrier of vesicles surrounding bacterial cells.

### 3.3. OMVs Associated with Resistant Bacterial Cell Do Not Sensitize to Phage Infection

**Figure 4:**
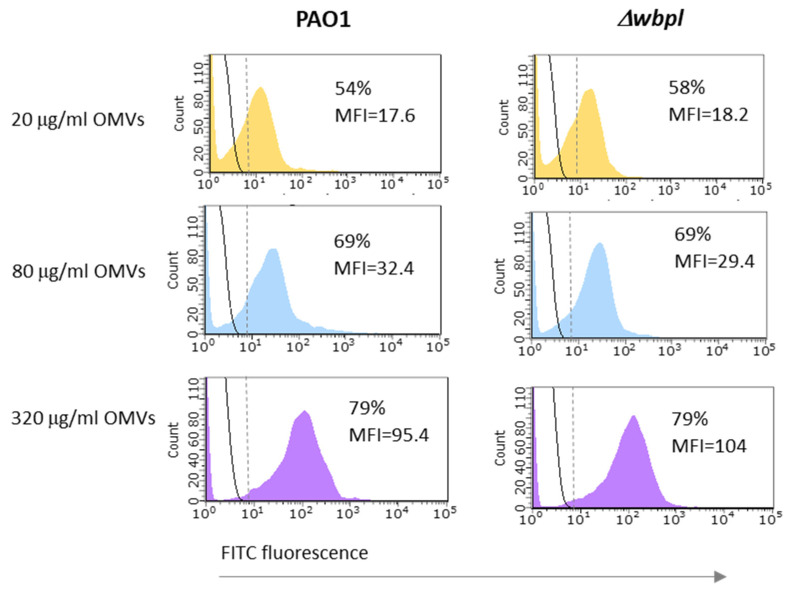
The association of OMVs to *P. aeruginosa* cells determined by the flow cytometry technique. The experiment was carried out on OMVs derived from PAO1. Bacteria were incubated with FITC-OMVs for 3 h at 37 °C with agitation (200 rpm). The fluorescence intensities of OMVs-associated bacteria are shown as coloured histograms, whereas control bacteria by black solid lines. Calculated percentages of OMV-associated cells include cells separated by a vertical dashed line. Data are expressed as mean fluorescent intensity (MFI) from a representative experiment.

The next part of the study was dedicated to the question of whether OMVs being associated with the bacterial cell can protect against the LPS-specific phages or, conversely, sensitize resistant bacteria to phage infection. To answer that, the PAO1 wild-type strain was used as the phage-sensitive host and its Δ*wbpl* knock-out mutant deficient in the A-band and B-band O-antigens as the phage-resistant strain. First, the association capability of OMVs was determined using flow cytometry (Figure 4). In this assay initially, various concentrations of OMVs were applied to find out the efficiency of cell-vesicle interactions and to check whether the entire population could be covered with FITC-labelled OMVs. As shown in Figure 4, there were no notable differences in association efficacy between PAO1 and the Δ*wbpl* mutant after 3 h of incubation. Although the intensity of association of OMVs was generally correlated with the number of vesicles available, even at their maximum tested amount (corresponding to a concentration of 320 μg/mL), an association of the entire bacterial population was not achieved. Both strains similarly interacted with OMVs at a level close to 80%.

**Figure 5:**
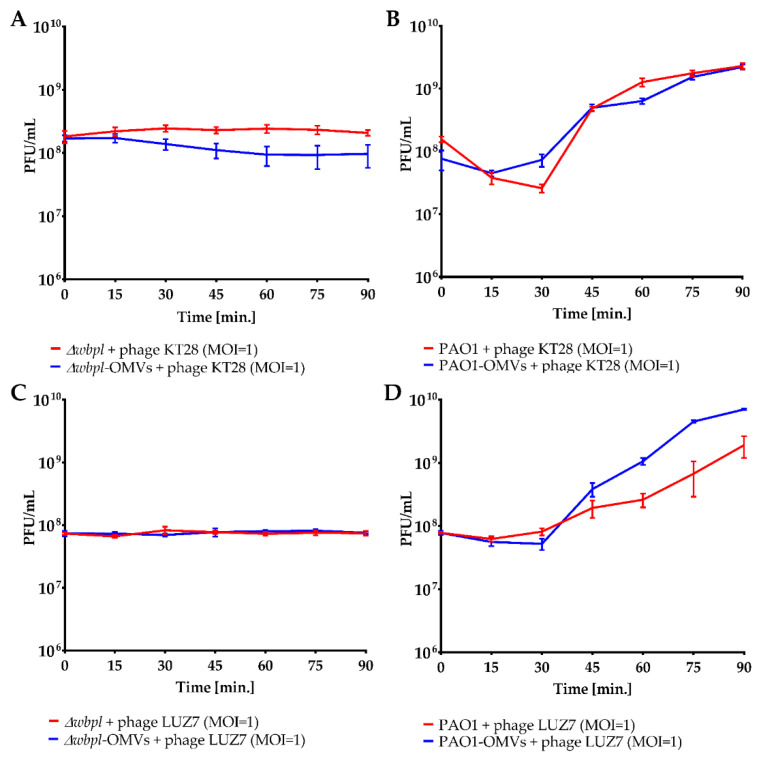
The OMVs association to *P. aeruginosa* cells as a potential sensitizer/disturbance agent to phage adsorption and propagation evaluated in a modified one-step growth cycle (MOI = 1). The PAO1 wild-type strain was used as the phage-sensitive host, whereas Δ*wbpl* knock-out mutant deficient in O-antigens served as the phage-resistant strain. Phage titre (PFU/mL) of myovirus KT28 (**A**,**B**) and podovirus LUZ7 (**C**,**D**) were determined every 15 min by the double-agar layer technique. The curves were established on the average of triplicate PFU/mL measurement from two independent experiments ± SEM bars.

To verify whether the cell-associated vesicles affect (positively or negatively) the phage adsorption and finally the propagation cycle, a modified one-step growth was assessed (Figure 5). As calculated by PFU counting, we could not detect the propagation of KT28 nor LUZ7 phages on the Δ*wbpl* mutant lacking O-antigens when associated with OMVs since it remained resistant to both phages, indicating a complete lack of sensitization (Figure 5A,C). On the other hand, verifying a potential adverse influence of OMVs association, we found that the wild-type PAO1 strain was still sensitive to KT28 and LUZ7 phages regardless of vesicle presence. The eclipse period was similar for OMV-associated and non-associated bacteria (Figure 5B,D).

**Figure 6:**
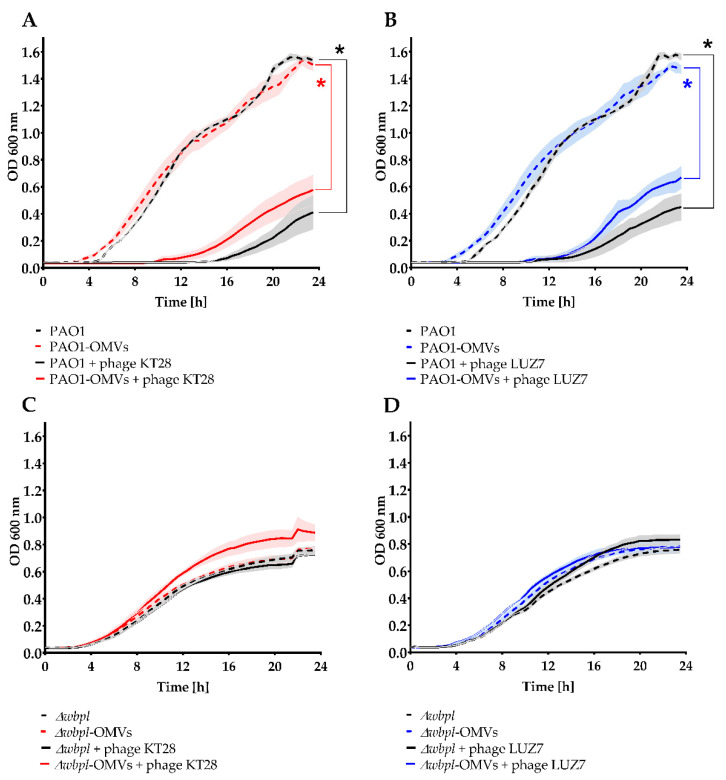
The OMVs association to *P. aeruginosa* cells as a potential sensitizer/disturbance agent to phage adsorption and propagation evaluated in bacterial culture growth kinetics. The PAO1 wild-type strain was used as the phage-sensitive host, whereas Δ*wbpl* knock-out mutant deficient in O-antigens served as the phage-resistant strain. Bacterial growth was monitored by OD_600_ measurement using a microplate reader for 24 h at 30 min intervals. The populations were treated with myovirus KT28 (**A**,**C**) or podovirus LUZ7 (**B**,**D**) at MOI = 1. The phage-uninfected culture without the addition of OMVs was considered as the control. The curves were established on the average of two independent experiments measured in triplicates ± SEM bars. Statistics were performed by Kruskal–Wallis ANOVA rang with pairwise AUC comparisons across groups: * *p* < 0.05.

The protection/sensitization potential of OMVs associated with bacteria was further investigated in the bacteria culture growth kinetics during 24 h of incubation, and individual curves were analysed based on the area under the curve (AUC) ± SEM (Figure 6). Growth curves for PAO1 and separately for the Δ*wbpl* mutant, after or without association showed similar characteristics, indicate that association does not interfere with cell multiplication. Analogous to the previous (one-step growth) experiment, the Δ*wbpl* mutant culture was not sensitized to lytic phage infection when associated with OMVs; thus, the bacteria could propagate freely regardless of KT28 or LUZ7 phages’ presence. On the contrary, the significant KT28-dependent growth inhibition of PAO1 (AUC = 2.48 ± 0.55 vs. AUC = 17.79 ± 0.16; *p* < 0.0005) and PAO1-OMVs (AUC = 4.91 ± 0.56 vs. AUC = 17.93 ± 1.06; *p* < 0.01) were demonstrated compared to bacteria cultured without phages. The analogous results were obtained for LUZ7-dependent growth inhibition of PAO1 (AUC = 2.64 ± 0.32 vs. AUC = 17.14 ± 0.32; *p* < 0.001) and PAO1-OMVs (AUC = 4.80 ± 0.53 vs. AUC = 17.85 ± 1.07; *p* < 0.005) when bacteria were not treated with phages. These results indicate that OMVs associated with phage-sensitive bacteria do not constitute a barrier against phage adsorption and propagation. However, although the PAO1-OMVs culture proved to be slightly less sensitive to both phages (starting MOI = 1) as compared to the sensitivity of non-associated PAO1, these differences were not statistically significant. The tendency of the faster emergence of phage resistant forms in the PAO1-OMVs growth curve might suggest that associated vesicles even gradually dissolved within dividing cells somehow affect the whole population, inducing phenotypic resistance to phages, thus indirectly protecting bacteria from infection.

## 4. Discussion

Membrane vesicles (MVs) are an extremely sophisticated bacterial secretion system, allowing the transport of a wide variety of compounds or structures and therefore facilitating rapid adaptation of the microbial population to changing environmental conditions or protection against adverse factors. Depending on their content (or composition of surface structures), OMVs can associate with the same or another cell of the same population of a competing species or even with eukaryotic host cells. OMVs contain both surface virulence factors (e.g., LPS, OMPs, iron and zinc acquisition systems) and specific cargoes, such as signalling molecules, enzymes, toxins, genetic elements, harmful metabolites, or other substances toxic to the bacterial cell. In summary, OMVs can have signalling, nutritional, offensive, or defensive functions [48].

In this study, we attempted to investigate the overall effect of OMVs on the interaction dynamics between two LPS-dependent bacteriophages (KT28 and LUZ7) and the *P. aeruginosa* population in the model of PAO1 strain. Several aspects have been analysed, such as the OMVs-bacteria interactions, the phage particles neutralization by OMVs, the passive protection against phage infection, and lastly a possible sensitization of phage resistant population. Confronting these complex issues, we performed a series of experiments using both the wild-type PAO1 strain and the Δ*wbpl* mutant incapable of O-antigen production. During all research procedures, only naturally produced OMVs by PAO1 (not acquired by mechanical or enzymatic cell damage) were used to mimic as closely as possible the common mechanisms occurring in bacterial culture. First, the adsorption ability of KT28 and LUZ7 phages on the surface of OMVs was tested using transmission electron microscopy and the virion infectivity and neutralization by the incubation of phages with the vesicles suspension. Next, the ability of *P. aeruginosa* cells to associate with OMVs was assessed using flow cytometry. The propagation of LPS-dependent phages on bacteria associated with OMVs and bacterial growth dynamics in the presence or absence of the phage were investigated. Finally, to dot the i’s, we tested whether the OMVs derived from PAO1 wild-type and associated with Δ*wbpl* PAO1 O-chain-deficient cells would transfer the receptors for KT28 and LUZ7 phages, enabling virus propagation on the sensitized deletant. At the outset of designing the experiments for this study, we deliberately abandoned the dual negative control of using OMVs isolated from the Δ*wbpl* strain. We considered it logical that vesicles produced by a strain lacking CPA/OSA could not sequester phage particles recognising the O-antigen. We found confirmation of our supposition in the work of Tzipilevich et al. [20], where this type of control was performed.

The results of the OMVs passive protection against phage infection left no illusions since vesicles protected the PAO1 population against both phages. However, we noted an interesting effect testing the neutralization of phage particles by OMVs. As a result, we observed that incubation with vesicles decreases the titre of myovirus KT28 only but did not affect the podovirus LUZ7 concentration.

The cloud of vesicles surrounding the bacteria is not just a physical veil because, as a piece of the outer bacterial membrane, it carries the receptors for phages (LPS, pili, OMPs). This allows phage particles to bind to OMVs surface, which might be followed by the ejection of viral genetic material into the vesicle interior, thus classified as an abortive infection (Abi) [19,41,42]. It should be emphasised that the vesicles containing the components of the bacterial periplasmic space might later be effectively fused with bacterial cells and potentially transfer phage genes into the new host [48].

The process of binding phages to bacterial surface structures is much more complicated and can provide permanent or reversible adsorption. Moreover, even if a given phage recognises and binds to a particular structure (e.g., the O-antigen of LPS), it does not mean the successful capsid content release or permanent neutralization of the phage. Firstly, there are different recognition capacities of phage tail spikes/fibres, and secondly, phages may require additional receptors (e.g., OMPs) for effective infection. In the case of *P. aeruginosa*, the O-antigen is produced in two variants as a common polysaccharide antigen (CPA, A-band), which is formed with repeated rhamnose residues, and as an O-specific antigen (OSA, B-band), which is composed of a wide variety of sugars [49,50]. These two types of LPS are produced by *P. aeruginosa* at different intensities depending on the population lifestyle (planktonic or sessile) [51]. Undoubtedly, it is difficult to say what ratio of CPA/OSA is present on the surface of the currently produced vesicle, but it would be relevant to LPS-dependent phages adsorption and eventually their inactivation. The previous report done on naturally produced OMVs by *P. aeruginosa* detected only the anionic OSA but not the neutral CPA presence [52]; thus, the receptor for both phages tested in our study was available in abundance in OMVs samples. A possibility that podovirus may be equipped with virion-associated O-chain depolymerase has been also taken into consideration as one of the factors differentiating the adsorption mechanism. Although SGNH hydrolase domain-containing tail fibre protein has been found in the phage LUZ7 genome, the lack of a characteristic halo zone surrounding phage LUZ7 plaques suggested no active depolymerase presence. The above aspects explain the discrepancies in the efficiency of phage particles interaction with purified LPS vs. OMVs [42] as well as the differences in KT28 and LUZ7 phages neutralization by *P. aeruginosa* OMVs in our studies.

The biogenesis of OMVs has not yet been thoroughly elucidated. Intensive research reveals new groups of molecules involved in the formation and release of vesicles, extending the three main models (related to lipoproteins, peptidoglycan, and LPS) that have been in existence for some time [53]. So far, we know that the main stimulator of OMVs production in *P. aeruginosa* is PQS (*Pseudomonas* quinolone signal), one of the key molecules of the complex quorum-sensing system of this bacterium. PQS, as a highly hydrophobic molecule, is transported to the outer membrane, where it changes the conformation of lipid A, resulting in membrane convexity [48,49,53,54,55,56,57,58]. In addition to lipid A, O-antigen is also involved in the formation of OMVs, and its electrical charge is crucial for the physical size and protein composition of OMVs [52,59]. Murphy et al. demonstrated that in the case of *P. aeruginosa*, proteomes of the OMVs produced by CPA-deficient mutants contain high amounts of proteins related to the transport of small-molecule substances. In contrast, the mutants possessing only uncharged A-band LPS produce OMVs accumulating proteins related to transcriptional regulation, adaptation, cell protection, and phage/plasmid/transposon handling [51,53]. It is therefore clear that the surface of OMVs produced by *P. aeruginosa* is heterogeneous in sugar, lipid, and protein content. The complex process of phage adsorption usually depends on more than one receptor, which may be some OM proteins; thus, once missing on the vesicle, the phage will not bind to it permanently.

Although we could not experimentally confirm the phenomenon of permanent neutralization of phage LUZ7 by OMVs, nevertheless, microscopic analysis confirmed that both the LPS-dependent phages tested in this study were adsorbed to vesicles, justifying the positive role of OMVs in passive protection against phage infection. The differences in the neutralization process might also depend on the dissimilarity of the DNA ejection mechanism present in podoviruses (phage LUZ7) vs. myoviruses (phage KT28).

MVs produced by bacteria (both gram-positive and gram-negative) usually contain receptors for phages. The possibility of transferring these receptors between different bacterial strains is therefore of great interest, as this could enable the sensitization of strains originally resistant to phages [60]. Encouraged by the work of Tzipilevich et al. [20], who demonstrated the existence of such a phenomenon in *Bacillus subtilis*, we tried to verify if it is possible to sensitize a CPA/OSA-deficient mutant (Δ*wbpl* PAO1) resistant to tested LPS-dependent phages by the association with OMVs isolated from a wild-type PAO1 strain. Documenting a quite effective association of OMVs to Δ*wbpl* mutant, firstly, we checked the growth curve of the culture with phage addition, and secondly, we measured the phage titre during a 90-min incubation with the bacterial cells associated with OMVs. Unfortunately, none of these experiments was successful since no sensitization was observed in contrast to previous reports published by other researchers [20]. Apart from the obvious difference in cell wall structure between gram-negative and gram-positive bacteria as well as completely different mechanisms of phage infection, the reason for our failure can be attributed to two main issues. First, unlike in the latter study, we did not co-culture a susceptible strain with a resistant strain but attempted to sensitise the O-antigen-deficient mutant with OMVs released from uninfected wild-type PAO1. This is important insofar as naturally produced vesicles differ in composition from those formed by lysis of phage-sensitive cells [42,61] or obtained by the French press procedure, as was done by other teams [42]. It is therefore quite possible that commonly formed OMVs did not contain the complete set of receptors for LPS-dependent phages (LUZ7 and KT28) and might not be able to transfer the susceptibility patterns to resistant cells. Finally, we cannot exclude the lack of potential fusion between the donor (OMVs) and the recipient (bacteria) cell membranes. Interestingly, to our knowledge, none of the published papers documenting a vesicle-dependent sensitization has experimentally proven the fusion event between OMVs and bacterial membranes [19,20,41,42].

The second problem arises from the association potential of vesicles, which can vary depending on the species of bacteria and the growth phase of the culture [62]. For example, during the incubation with OMVs (80 µg/mL), *Moraxella catarrhalis* can associate more than 90% of the OMVs [18], whereas our measurements in *P. aeruginosa* revealed an association at the level of ~70%. Thus, even if the transfer of receptors was effective, only a part of the population received them, and likely, the number of vesicles associated with the OM of individual bacteria was limited.

Although the idea of OMVs role as the phage receptor vehicle to sensitize resistant bacteria is very tempting, we should remember that from the co-evolutionary point of view both bacteria and phages have developed specific strategies/mechanisms to avoid the susceptibility overload to viral infection in order to protect the resources of both parties.

Considering the future directions, a very interesting issue seems to be the influence of the prophages on the intensity of secretion and the composition of released OMVs. So far, we used only the prophage-free *P. aeruginosa* PAO1 strain. In future studies, we will certainly use prophage-carrying strains to determine whether a lysogenic strain producing vesicles can protect strains lacking prophages against phage infection in the superinfection exclusion (Sie) phenomenon [63].

## Figures and Tables

**Table 1:** Characteristics of phages used in this work.

Phage	Taxonomy (Family, Genus)	Genome Size	GenBank	Recognized Bacterial Receptor	Reference
KT28 **	*Myoviridae*, *Pbunavirus*	66,381 bp	KP340287	LPS	[43]
LUZ7 *	*Schitoviridae*, *Luzseptimavirus*	74,901 bp	NC_013691	LPS	[44]

* Laboratory of Gene Technology, KU Leuven, Leuven, Belgium. ** Department of Pathogen Biology and Immunology, Institute of Genetics and Microbiology, University of Wroclaw, Wroclaw, Poland.

## Data Availability

Not applicable.

## Supplementary Materials

The following supporting information can be downloaded at: https://www.mdpi.com/article/10.3390/v14010121/s1, Figure S1: The inhibition of the lytic activity of myovirus KT28 against *P. aeruginosa* PAO1 (MOI = 1) in the presence of various concentrations of OMVs from PAO1: (A) An assay for phage particles neutralization on OMVs surface determined by PFU/mL counts. The phage-uninfected PAO1 culture without the addition of OMVs was considered as the control. (B) The OMV-mediated passive protection assay of PAO1 cells against phages determined by CFU/mL counts. The representative experiments show mean CFU/mL or PFU/mL ± SEM bars.
