# Supplementary material for: Outer Membrane Vesicles (OMVs) of Pseudomonas aeruginosa Provide Passive Resistance but Not Sensitization to LPS-Specific Phages
Source: Viruses. 2022 Jan 11;14(1):121. doi: 10.3390/v14010121 (PMC8778925; doi:10.3390/v14010121)
Supplement: Supplementary file 1 [file viruses-14-00121-s001.zip › viruses-1514162-supplementary.pdf]

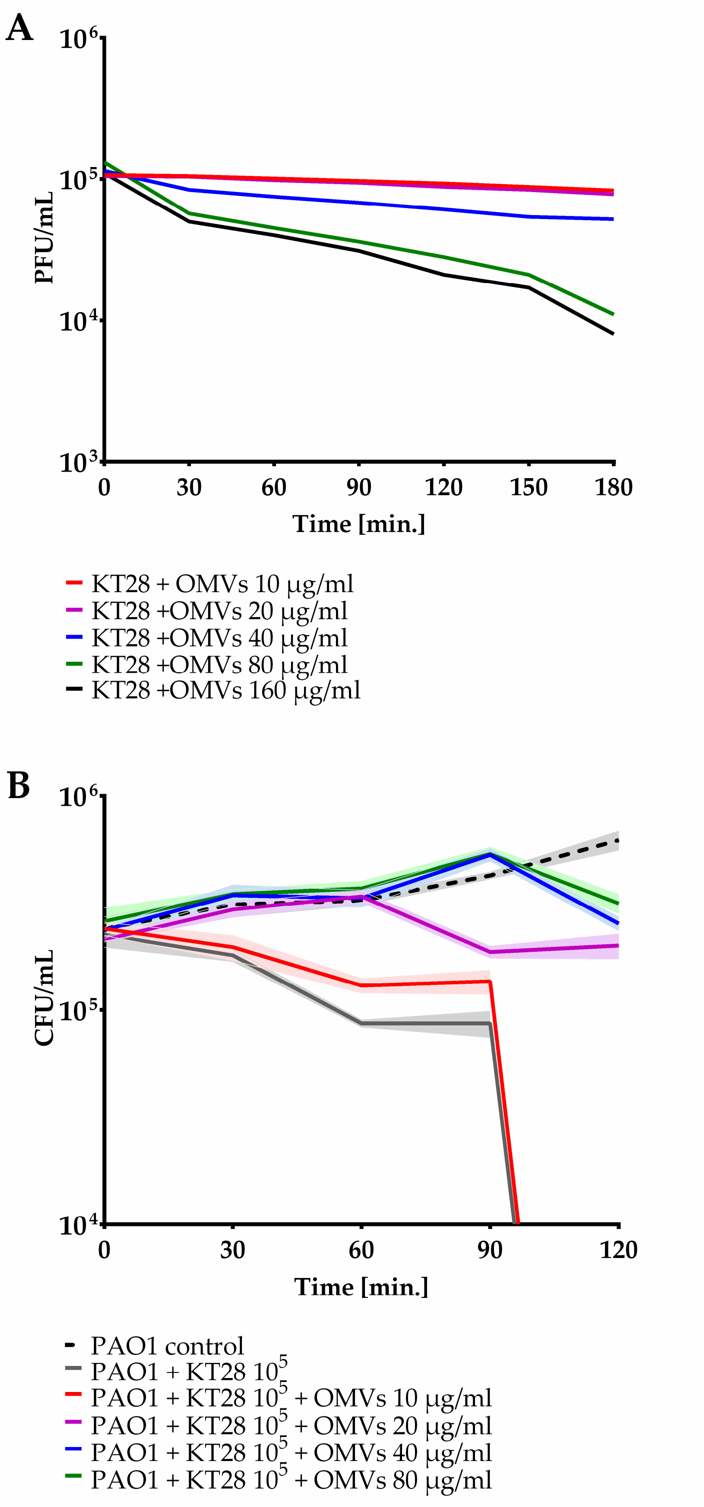

Figure S1. The inhibition of the lytic activity of myovirus KT28 against *P. aeruginosa* PAO1 (MOI = 1) in the presence of various concentrations of OMVs from PAO1: (A) An assay for phage particles neutralization on OMVs surface determined by PFU/mL counts. The phage-uninfected PAO1 culture without the addition of OMVs was considered as the control. (B) The OMV-mediated passive protection assay of PAO1 cells against phages determined by CFU/mL counts. The representative experiments show mean CFU/mL or PFU/mL ± SEM bars.
